# Systematic Review of Patient-Derived Xenograft Models for Preclinical Studies of Anti-Cancer Drugs in Solid Tumors

**DOI:** 10.3390/cells8050418

**Published:** 2019-05-06

**Authors:** Yoshikatsu Koga, Atsushi Ochiai

**Affiliations:** 1 Department of Strategic Programs, Exploratory Oncology Research & Clinical Trial Center, National Cancer Center, Kashiwa 277-8577, Japan; 2 Exploratory Oncology Research & Clinical Trial Center, National Cancer Center, Kashiwa 277-8577, Japan; aochiai@east.ncc.go.jp

**Keywords:** PDX clinical trial, co-clinical trial, drug development, solid tumor, patient-derived cancer model, drug sensitivity

## Abstract

Patient-derived xenograft (PDX) models are used as powerful tools for understanding cancer biology in PDX clinical trials and co-clinical trials. In this systematic review, we focus on PDX clinical trials or co-clinical trials for drug development in solid tumors and summarize the utility of PDX models in the development of anti-cancer drugs, as well as the challenges involved in this approach, following the preferred reporting items for systematic reviews and meta-analyses (PRISMA) guidelines. Recently, the assessment of drug efficacy by PDX clinical and co-clinical trials has become an important method. PDX clinical trials can be used for the development of anti-cancer drugs before clinical trials, with their efficacy assessed by the modified response evaluation criteria in solid tumors (mRECIST). A few dozen cases of PDX models have completed enrollment, and the efficacy of the drugs is assessed by 1 × 1 × 1 or 3 × 1 × 1 approaches in the PDX clinical trials. Furthermore, co-clinical trials can be used for personalized care or precision medicine with the evaluation of a new drug or a novel combination. Several PDX models from patients in clinical trials have been used to assess the efficacy of individual drugs or drug combinations in co-clinical trials.

## 1. Introduction

Cancer is the leading cause of death in economically developed countries, and many physicians and scientists are using their efforts to develop new treatment approaches. At present, surgery, radiation therapy, and chemotherapy are the three pillars for curing and prolonging survival of patients with cancer. Among these treatments, cancer chemotherapy plays a central role for patients with advanced or recurrent cancer. Conventional anti-cancer drugs can directly kill rapidly proliferating cells, while small molecule inhibitors and therapeutic antibodies can inhibit the intracellular growth signal cascade and lead to cancer cell-specific death [1,2]. For the development of anti-cancer drugs, in vitro cell killing assays using commercially available patient-derived cell lines or in vivo tumor growth inhibition assays using cell-line-derived xenograft (CDX) models are commonly employed to measure the efficacy of drugs and to make a “go or no-go” decision for further clinical study. Unfortunately, few drugs are approved even if the drugs demonstrate a good response in preclinical studies. Indeed, only 5% of the anti-cancer drugs that have anti-cancer activity in preclinical studies are approved for clinical application by the United States Food and Drug Administration (FDA) [3]. 

To develop anti-cancer drugs for solid tumors, knowledge of the hallmarks of cancer and the cancer microenvironments is important [4]. The cancer microenvironments consist of cancer cells and the surrounding cancer stromal cells. These stromal cells, including tumor endothelial cells (TECs) [5], cancer-associated fibroblasts (CAFs) [6], and tumor-associated macrophages (TAMs) [7], are educated and activated by growth factors produced from cancer cells and promote cancer progression and metastasis. Additionally, the cancer stromal cells produce a collagen-rich extracellular matrix (ECM), which can interrupt drug distribution in the cancer tissue. Thus, knowledge of the cancer stroma is important for the development of drugs targeting solid tumors. 

Traditional CDX models consist of many cancer cells but few cancer stromal cells, and they are difficult to use in preclinical models for predicting the response in clinical trials. This prompted attempts to inject patient-derived cancer tissue into immunodeficient mice, which has been conducted for over 40 years. These patient-derived xenograft (PDX) models conserve the biological features (histological architecture, especially cancer stroma construction, and gene-expression or mutation status) of the original tissue. A significant association was observed between drug responses in patients and the corresponding PDX models in 87% (112/129) of therapeutic outcomes. Thus, PDX models are recognized as accurate and clinically relevant models [8]. The National Cancer Institute (NCI)-60 panel, which contained 60 human cancer cell lines, was heavily used by researchers around the world for anti-cancer drug screening for over 30 years. In 2016, the United States NCI decided to stop screening of anti-cancer drugs using the NCI-60 panel and focus on newer PDX models [9]. 

There are several global PDX repositories. The EurOPDX consortium, which was launched in 2013 and consists of 18 European and US institutions, has established more than 1500 PDX models for more than 30 pathologies (https://www.europdx.eu/). CrownBio (more than 2500 PDX models, https://www.crownbio.com/), Champions Oncology (more than 1000 PDX models, https://championsoncology.com/), the Jackson Laboratory (more than 400 PDX models, https://www.jax.org/), and DNA Link (more than 300 PDX models, http://www.pdx.dnalink.com/index) are major contract research outsourcing companies for PDX models (the list of PDX providers is far from complete). Additionally, the Novartis Institutes for BioMedical Research PDX encyclopedia (NIBR PDXE) contains more than 1000 PDX models [10]. Using these large repositories, PDX models have been used for PDX clinical trials in preclinical studies for clinical decision making. PDX clinical trials are important for the development of anti-cancer drugs prior to clinical trials. 

Co-clinical trials are run in parallel and real-time with human clinical trials, and they include mouse trials using PDX models established from the clinical trial participants for assessment of drug response. This approach is recognized as a model for personalized care or precision medicine [11,12]. PDX models used in a co-clinical trial are also called “avatar” or “mirror” models. Several researchers have reported a high rate of drug response concordance in patients and their PDX models [13,14], indicating that these models can function as “mirror” models for the donor patients. On the other hand, a PDX model can be treated not only with the same drug used in the donor patient but also with other drugs or a novel drug combination. The PDX model, in this case an “avatar” model, can predict both the development of resistance to first-line therapy as well as the response to second-line therapy before these events are observed in the donor patient [15]. 

To date, PDX models have been used as powerful tools for understanding cancer biology, as a conventional assay for drug sensitivity or drug delivery, in PDX clinical trials before early clinical trials in humans, and in co-clinical trials. These models are also used in basic research for academia as well as in preclinical research for pharmaceutical companies. In this systematic review, we focus on PDX clinical trials or co-clinical trials for drug development in solid tumors and summarize the utility of and concerns associated with the use of PDX models for the development of anti-cancer drugs.

## 2. Materials and Methods

This study was reported according to the preferred reporting items for systematic review and meta-analysis (PRISMA) statement [16].

### 2.1. Article Search Strategy

Although the aim of this study was to identify the benefits and challenges associated with PDX models for preclinical study or co-clinical study, some articles using PDX models were not searched and this review is done at the study level. Articles were searched on 23 January, 2019 by PubMed online (https://www.ncbi.nlm.nih.gov/pubmed/) with the terms “PDX clinical trial” or “patient-derived xenograft clinical trial”, thus, the articles of PDX models used for the research of tumor biology and/or drug development were not searched completely. In fact, articles regarding “PDX clinical trials” or “co-clinical trials” are discussed in this review. All articles reviewed in this study were published after January 2010. 

### 2.2. Eligibility Criteria

The inclusion criteria for the articles in this review are as follows: original articles, written in English, available in full text, solid tumor PDX models, and human PDX models. Simulation studies, review articles, articles using leukemia or lymphoma PDX models, and articles only using cell lines or CDX models are excluded from this review. 

### 2.3. Study Selection and Data Collection

All articles were obtained as full paper copies and were evaluated to determine if the eligibility criteria of this review were met. The data extracted from the full papers were as follows: publication year, cancer type (origin), mouse strain, implantation site, application for which the PDX model was used, number of the PDX model, and the treatment drug. 

## 3. Results

**Figure 1:**
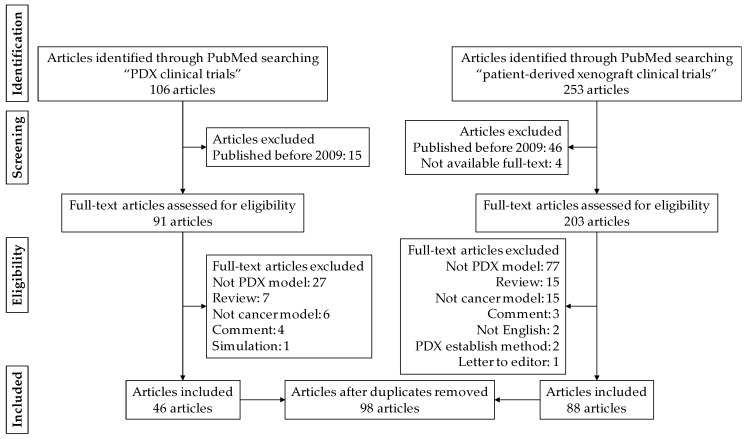
Preferred reporting items for systematic reviews and meta-analyses (PRISMA) flow diagram of the article selection process.

Article selection by a systemic review process [16] was as presented in Figure 1. A total of 106 articles and 253 articles were identified through PubMed search, using the terms “PDX clinical trial” and “patient-derived xenograft clinical trial”, respectively. To investigate the recent use of PDX models, 61 articles were excluded because these articles were published before 2009. Four articles for which full-texts were not available were also excluded during the screening phase. Among 294 full-text articles, 160 articles were excluded after reading the full text. The main reasons were that the article did not include a PDX model, meaning not a solid tumor PDX model, not a human PDX model, or PDX was used to refer to something other than the abbreviation for patient-derived xenograft, e.g., polydextrose. Since 36 articles were duplicates, the final number used for this review was 98 articles. 

### 3.1. Publication Year

**Figure 2:**
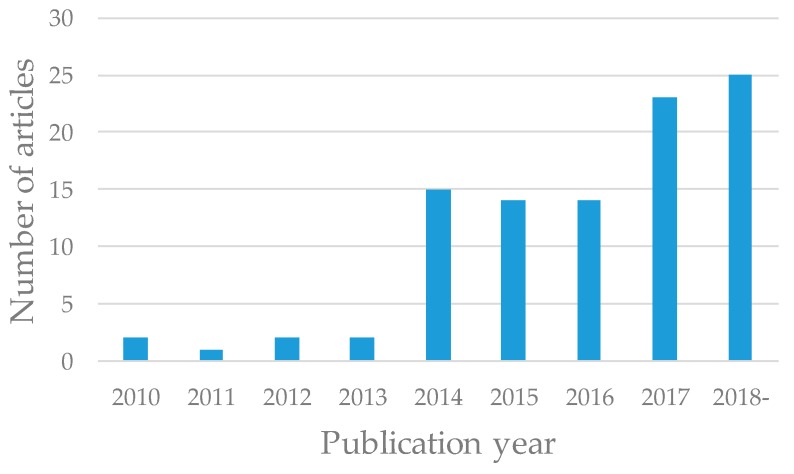
Number of articles published in each year.

The first PDX model, in which human sigmoid colon cancer tissues were inoculated subcutaneously into nude mice, was reported in 1969 [17]. Since then, PDX models have been reported in numerous articles. We selected PDX models employed for clinical use in this review. The publication history of papers describing these studies is shown in Figure 2. A few articles were published per year before 2013, two articles were published in 2010 [18,19], one article in 2011 [20], two articles in 2012 [21,22], and two articles in 2013 [23,24]. On the other hand, more than 10 articles were published each year after 2014, 15 articles were published in 2014 [25,26,27,28,29,30,31,32,33,34,35,36,37,38,39], 14 articles in 2015 [10,40,41,42,43,44,45,46,47,48,49,50,51,52], 14 articles in 2016 [53,54,55,56,57,58,59,60,61,62,63,64,65,66], 23 articles in 2017 [67,68,69,70,71,72,73,74,75,76,77,78,79,80,81,82,83,84,85,86,87,88,89], and 25 articles during 2018 or after [15,90,91,92,93,94,95,96,97,98,99,100,101,102,103,104,105,106,107,108,109,110,111,112,113]. In 2011, Bertotti et al. reported the efficacy of cetuximab using 85 PDX models of patients with metastatic colorectal cancer, demonstrating the utility of PDX models for preclinical studies [20]. The number of articles describing PDX models in preclinical applications has been increasing over the past several years. To assess the frequency of PDX models used in preclinical settings, 98 articles were divided into two periods, those published from 2010 to 2016 (50 articles) and during or after 2017 (48 articles). 

### 3.2. Types of Cancers Used for PDX Models

**Figure 3:**
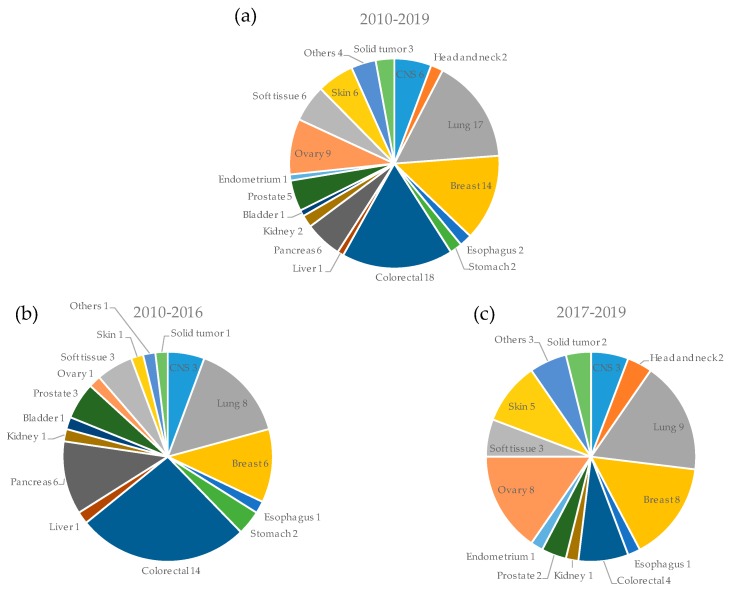
Cancer types used for patient-derived xenograft (PDX) models. The number of PDX models for each organ is shown for all periods (**a**), the first period from 2010 to 2016 (**b**), and the second period from 2017 onwards (**c**).

The types of cancers used for PDX models are summarized in Figure 3. During the overall study period, major cancers, such as colorectal cancer (18%, 18/99) [19,20,21,29,41,42,47,48,49,51,52,56,58,60,79,87,108,111], lung cancer (17%, 17/99) [18,25,35,40,43,44,49,64,71,72,73,78,84,104,106,110,112], and breast cancer (14%, 14/99) [26,28,46,55,61,65,71,77,85,86,88,89,91,102] were used more frequently for PDX models. The others were ovarian cancer (9%, 9/99) [49,67,70,71,80,95,97,100,101], brain tumors (6%, 6/99) [57,59,63,83,101,103], pancreatic cancer (6%, 6/99) [22,24,27,39,45,54], sarcoma (6%, 6/99) [23,32,37,75,99,109], melanoma (6%, 6/99) [31,76,93,94,96,98], prostate cancer (5%, 5/99) [34,53,66,92,105], head and neck cancer (2%, 2/99) [90,107], esophageal cancer (2%, 2/99) [38], gastric cancer (2%, 2/99) [33,65], renal cancer (2%, 2/99) [30,67], bladder cancer (1%, 1/99) [50], endometrial cancer (1%, 1/99) [74], hepatoblastoma (1%, 1/99) [62], and others (adenoid cystic carcinoma [69,82], solitary fibrous tumor [36], clear cell adenocarcinoma [15]). In the PDX clinical trials or co-clinical trials, solid tumor including several cancers were used [10,81,113]. When comparing both time periods (2010–2016 and 2017 onward), we noted that the use of PDX models in ovarian cancer and melanoma increased (first period:second period, 2%:16% for ovarian cancer, and 2%:10% for melanoma). On the other hand, the use of PDX models in colorectal cancer and pancreatic cancer is decreasing (26%:8% for colorectal cancer, and 11%:0% for pancreatic cancer). Recently, the diversity of cancer types used has been increasing. 

### 3.3. Mouse Strain and Implantation Site for PDX Model

**Figure 4:**
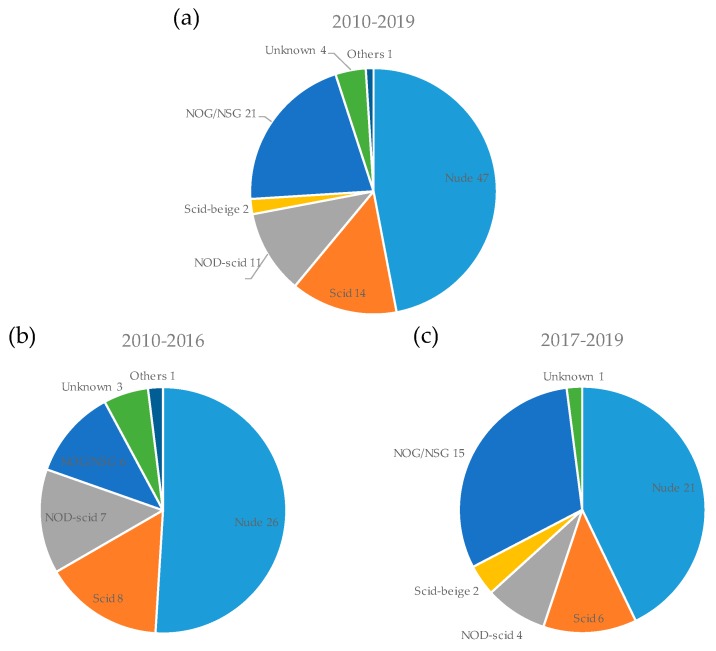
Mouse strains used for PDX models. The number of PDX models for each mouse strain is shown for all periods (**a**), the first period from 2010 to 2016 (**b**), and the second period from 2017 onwards (**c**).

The mouse strains used for PDX models are shown in Figure 4. A PDX model can be developed by implantation of cancer tissue into an immunodeficient mouse. Although a variety of mouse strains were used, the most common strains were nude (47%, 47/100), scid (14%, 14/100), NOD-scid (11%, 11/100), and NOG/NSG (the same as NOD/scid/IL2γ-receptor null; 21%, 21/100). Nude mice lack mature T cells, scid mice lack mature B and T cells, NOD-scid mice lack mature B and T cells and have impaired NK cells, and NOG/NSG mice lack mature B and T cells, as well as NK cells [114]. Two other immunodeficient mouse strains, scid-beige [70,101] and RAG2 null/γC null [30], were also used. The use of NOG/NSG mice is increasing because these are most severely immunodeficient and the tumor engraftment rate (take-rate) is the best among the mouse strains. However, nude mice are still the most commonly used strain, owing to their low cost and because hairless mice are easy to assess for tumor growth. Recently, several cancer immunotherapies have been assessed in preclinical and clinical studies. Conventional PDX models are difficult to use to analyze the efficacy of immuno-oncology drugs, as the immunodeficient mice have no cytotoxic T cells. Therefore, syngeneic mouse models are commonly used for the development of these drugs. To resolve this issue, humanized PDX models have been developed. Vargas et al. reported on humanized PDX models used to evaluate treatment with the immune checkpoint inhibitor nivolumab using NSG mice with an injection of tumor tissue as well as the patient’s peripheral blood lymphocytes (PBLs) [15]. 

**Figure 5:**
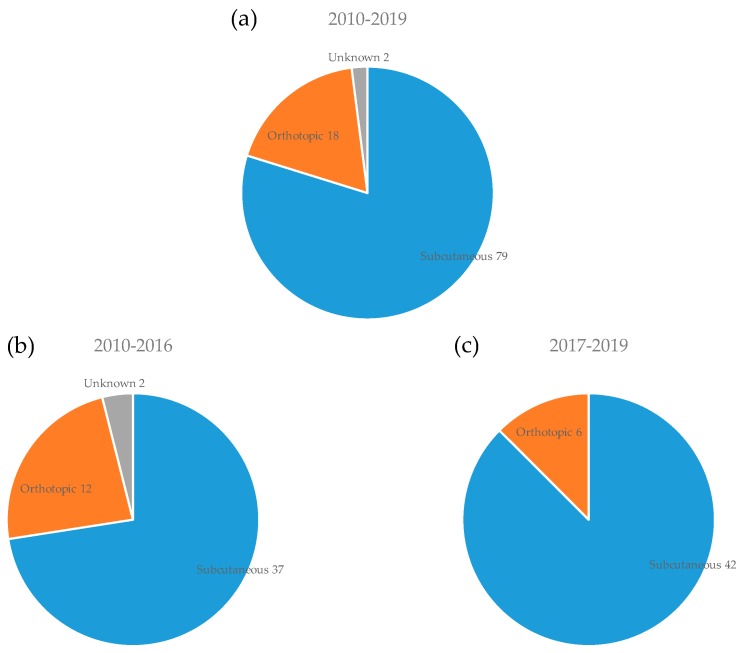
Implantation sites of cancer tissues for PDX models. The number of PDX models for each implantation site is shown for all periods (**a**), the first period from 2010 to 2016 (**b**), and the second period from 2017 onwards (**c**).

The tumor implantation sites are shown in Figure 5. Although subcutaneous implantation was most common (80%, 79/99), orthotopic implantation was sometimes selected for several types of cancer, including the implantation of primary or metastatic brain tumor into the brain [57,61,63,83,101], breast cancer into mammary gland [26,46,91], and renal cancer into renal capsule [30]. Although the patient-derived orthotopic xenograft (PDOX) models mimic metastasis of cancer and are important models for basic and applied research compared to PDX models [115], the subcutaneous PDX models are commonly used in the PDX clinical trials or co-clinical trials because the PDX models are easy to assess the drug efficacy compared to the PDOX models.

### 3.4. Applications of PDX Models in Cancer Research

**Figure 6:**
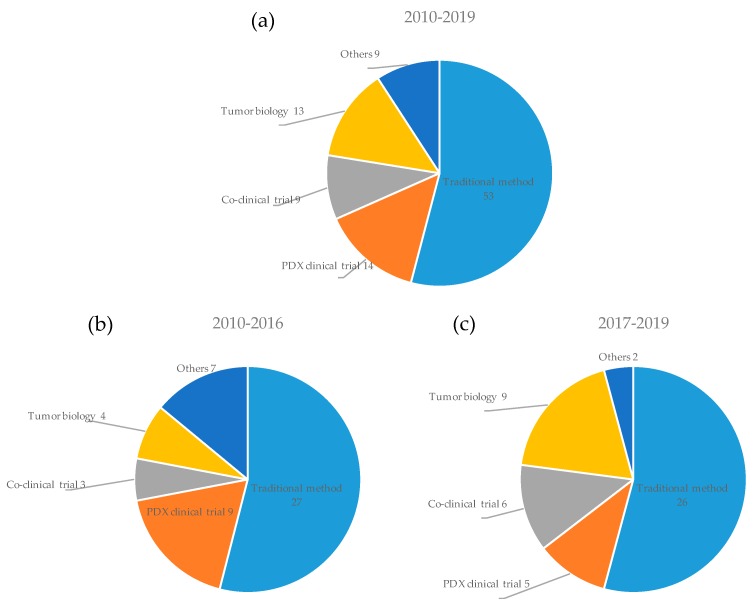
Application of PDX models in cancer research. The number of PDX models for each application is shown during all periods (**a**), the first period from 2010 to 2016 (**b**), and the second period from 2017 onwards (**c**).

Applications of PDX models in cancer research are shown in Figure 6. The efficacy of potential anti-cancer drugs can be investigated using cell lines in vitro and CDX models in vivo. In addition, PDX models can then be used to conduct a drug efficacy study. The majority of articles were categorized into this traditional method (54%, 53/98). An article representative of this method was published by Cho et al. in 2018 [92]. In the study, the efficacy of anti-prostate-specific membrane antigen (PSMA) antibody drug conjugate (ADC) was assessed using prostate cancer models. First, the IC_50_ of the ADC was analyzed using six cell lines with several PSMA expression levels. Next, tumor growth inhibition by ADC administration was assessed in three CDX models with several PSMA expression levels. Finally, the efficacy of the ADC was assessed in eight PDX models with several PSMA expression levels. The PSMA-ADC demonstrated potent and specific cytotoxicity in PSMA-positive cell lines, and robust and durable antitumor activity in PSMA-positive CDX and PDX models. Drug efficacy was also assessed by PDX clinical trials and co-clinical trials as summarized in the following two sections.

Tumor biology is also an important subfield of cancer research. Bonanno et al. clarified the relationship of liver kinase B1 (LKB1) expression level with sensitivity to bevacizumab using PDX models [68]. Bertolini et al. showed that CD133^+^/CXCR4^+^ cancer initiating cells isolated from PDX models had a superior ability to seed and initiate metastasis at distant organs [40]. Comparing the 2010-2016 period to 2017 onwards shows that the percentage of studies investigating mechanisms or biomarkers for cancer growth or metastasis using PDX models has doubled (first period:second period, 8%:19%). 

### 3.5. PDX Clinical Trials

Fourteen articles reporting on PDX clinical trials are summarized in Table 1. PDX clinical trials are important for the development of anti-cancer drugs and clinical decision making before human clinical trials. This is reflected in the fact that 20 to 30 PDX models were enrolled in most of the PDX clinical trials. As PDX clinical trials are also referred to as “phase II type clinical trial like models”, several solid tumors were enrolled following an “all-comer model” [10]. The tumor growth in PDX models in such PDX clinical trials have commonly been assessed by the modified response evaluation criteria in solid tumors (mRECIST) guidelines and shown in a waterfall plot as is done in human clinical phase II trials. During the first study period, five to 10 animals per treatment group were commonly used. In 2015, Gao et al. reported a high-throughput in vivo drug screening method using a large number of PDX models [10]. The study design was “1 × 1 × 1”, which meant one animal per model per treatment. After the publication of this article, 1 × 1 × 1 approaches were mainly used in PDX clinical trials. However, as one animal per PDX model might not be representative of generalizable drug response, two or three animals per model per treatment (2 × 1 × 1 or 3 × 1 × 1) PDX clinical trials have recently become more common. 

### 3.6. Co-clinical Trials

Nine articles described co-clinical trials. These are summarized in Table 2. Co-clinical trials are recognized as “avatar” models for personalized care or precision medicine [11,12]. Thus, the number of PDX models enrolled in most co-clinical trials was less than 10. Since the biopsy specimens collected from metastatic lesion were small, NOG/NSG mice were used in half of the co-clinical trials to increase the engraftment rate. Unlike PDX clinical trials, five to 10 animals per treatment group were used in most of these co-clinical trials. PDX models using co-clinical trials were established from patients enrolled in clinical trials, and most of the cancer tissues were collected prior to treatment. Stebbing et al. reported a correlation between PDX model results and clinical outcomes (81%, 13/16) [37]. Frankel et al. reported that responses to anti-cancer drugs in a PDX model reflected the responses in patients [76]. The PDX model reported by Campbell et al. was generated from a patient treated with a mitogen-activated protein kinase kinase (MEK, also known as MAPKK) inhibitor for two weeks in a neoadjuvant setting [90]. Although the patient had a clinical response with down-staging of the tumor and underwent surgical resection, the patient later developed metastasis. In the co-clinical trial, the PDX model was treated with a longer course of the MEK inhibitor to determine whether this regimen had a clinical benefit. In this case, the MEK inhibitor suppressed tumor growth of the PDX model over the first 50 days of treatment; however, the tumor re-grew after 50 days. Thus, the PDX model displayed responses consistent with the clinical findings in the patient. In some co-clinical trials, not only were the same anti-cancer drugs used in clinical trials evaluated, but novel drugs or combinations were also assessed [81]. Interestingly, Vargas et al. reported a case study of real-time personalized care in a co-clinical trial [15]. In the case study, the response to first- and second-line therapies in PDX models could predict the response to first-line therapy, development of resistance and response to second-line therapy before these events were observed in the patient. 

## 4. Discussion

Since the articles were searched using the terms “PDX clinical trial” or “patient-derived xenograft clinical trial”, some articles using PDX models were not identified and this review is done at the study level. Cell lines or CDX models used for drug screening fail to accurately predict the clinical efficacy of anti-cancer drugs. Thus, PDX models are powerful preclinical models for cancer research, especially drug development, in several cancer types [116]. Although major cancers were often used for PDX models, the need for rare cancer PDX models for use in PDX clinical trials or co-clinical trials is increasing [15]. Regarding pediatric cancer, US Pediatric Preclinical Testing Consortium (http://www.ncipptc.org/) and Children’s Oncology Group cell culture and xenograft repository (https://www.cccells.org/index.php) have a lot of PDX models of rhabdomyosarcoma, Ewing sarcoma, neuroblastoma, and osteosarcoma, and are contributed to drug development. The PDOX models are commonly used for neuroblastoma because the tumors spontaneously metastasize to the liver, lung and bone marrow and are promising models for studying and targeting human neuroblastoma metastases [117,118]. The other topic of PDX model is humanized PDX model for the development of immuno-oncology (IO) drugs [119]. The conventional PDX models are lack of mouse immune systems and human immune cells, thus, IO drug, such as immune checkpoint inhibitor, is difficult to be assessed by using these models. To resolve this issue, PBLs or tumor-infiltrating lymphocytes (TILs) are injected into NOG/NSG mice [15]. However, these procedures cause severe graft-versus-host disease within two to five weeks after injection of PBL or TIL, thus, these mice are used only for short-term assessing of IO drugs. Compared to PBL or TIL mice, the models of transplantation of CD34-positive human hematopoietic stem cells (HSCs) isolated from umbilical cord blood into NOG/NSG mice have complete hematopoietic reconstitution. These next-generation PDX models are used for the development of IO drugs. 

The average engraftment rate of PDX models was recently estimated at 49% [8], allowing the PDX model to be commonly used not only for basic cancer research but also for assessing drug efficacy in preclinical research. However, the resources and processes for creating and characterizing PDX models differ greatly among academic institutions. To address this, Meehan et al. published the PDX models minimal information standard (PDX-MI) for reporting on the generation, quality assurance, and use of PDX models. This standard was developed by four independent PDX model resources (the EurOPDX consortium, the IMODI consortium, the Patient-Derived Models Repository at NCI-Federick, and the Jackson Laboratory PDX Resource) [120]. For consistency, PDX models should be reported and generated according to this standard. 

The traditional PDX models have commonly been used for the development of anti-cancer drugs. PDX clinical trials and co-clinical trials using PDX models are becoming an increasingly important method for drug development. PDX clinical trials, such as “phase II type clinical trial like mouse models”, can be used for the development of anti-cancer drugs before human clinical trials, and the results assessed by the mRECIST guidelines can be shown in a waterfall plot. In these studies, a few dozen PDX models from a PDX repository are enrolled and the efficacy of the drugs is assessed by 1 × 1 × 1, 2 × 1 × 1 or 3 × 1 × 1 approaches. On the other hand, co-clinical trials, such as “avatar” models, can be used for personalized care or precision medicine for the evaluation of a new drug or a novel combination. In these studies, several cases of PDX models from patients in clinical trials are used to determine the efficacy of the drugs or drug combinations.

## 5. Conclusions

PDX models are useful preclinical models for the development of anti-cancer drugs employed in PDX clinical trials and co-clinical trials. Information regarding the generation, quality assurance, and use of PDX models should be recorded according to the PDX-MI. 

## Figures and Tables

**Table 1:** Summary of PDX clinical trials.

Author	Year^1^	Origin^2^	Cases^3^	Strain^4^	Site^5^	Animals^6^
Hammer [18]	2010	NSCLC^7^	22	Nude	sc^12^	6
Bertotti [20]	2011	CRC^8^	85	NOD-scid	sc^12^	6
Laheru [22]	2012	Pancreas	14	Nude	sc^12^	5
Amendt [25]	2014	NSCLC^7^	45	Nude	sc^12^	10
Chen [41]	2015	CRC^8^	27	Nude	sc^12^	5
Gao [10]	2015	Solid tumor	1075	Nude	sc^12^	1
Pan [50]	2015	Bladder	22	NSG	sc^12^/ortho^13^	8 to 10
Guo [56]	2016	CRC^8^	25	NSG	sc^12^	Unknown
Gupta [57]	2016	GBM^9^	28	Nude	ortho^13^	8 to 10
Bialucha [67]	2017	Ovary/RCC^10^	30	Nude	sc^12^	1
Yao [87]	2017	CRC	79	Nude	sc^12^	1
Einarsdottir [96]	2018	Melanoma	31	NOG	sc^12^	1
Ruicci [107]	2018	HNSCC^11^	20	NSG	sc^12^	2
Zhong [113]	2019	Breast/Ovary	23	Nude	sc^12^	2 to 3

^1^ Publication year. ^2^ Origin of cancer for PDX models. ^3^ Number of PDX models. ^4^ Mouse strain. ^5^ Implantation site. ^6^ Number of animals per each treatment group. ^7^ Non-small cell lung cancer. ^8^ Colorectal cancer. ^9^ Glioblastoma. ^10^ Renal cell carcinoma. ^11^ Head and neck squamous cell carcinoma. ^12^ Subcutaneously implantation. ^13^ Orthotopic implantation.

**Table 2:** Summary of co-clinical trials.

Author	Year^1^	Origin^2^	Cases^3^	Strain^4^	Site^5^	Animals^6^
Stebbing [37]	2014	Sarcoma	16	Nude	sc^11^	Unknown
Kopetz [48]	2015	CRC^7^	1	NSG	sc^11^	1
Owonikoko [64]	2016	SCLC^8^	5	Nude	sc^11^	3 to 6
Frankel [76]	2017	Melanoma	4	NSG	sc^11^	7 to 10
Kim [78]	2017	LSCC^9^	5	NOG/Nude	sc^11^	6 to 7
Pauli [81]	2017	Solid tumor	19	Nude	sc^11^	5
Campbell [90]	2018	OCSCC^10^	1	NSG	sc^11^	7
Harris [98]	2018	Melanoma	3	Nude	sc^11^	8 to 10
Vargas [15]	2018	Clear cell adenocarcinoma	1	NSG	sc^11^	3

^1^ Publication year. ^2^ Origin of cancer for PDX models. ^3^ Number of PDX models. ^4^ Mouse strain. ^5^ Implantation site. ^6^ Number of animals per each treatment group. ^7^ Colorectal cancer. ^8^ Small cell lung cancer. ^9^ Lung squamous cell carcinoma. ^10^ Oral cavity squamous cell carcinoma. ^11^ Subcutaneously implantation.
